# Assessing the Probiotic Effects of *Pediococcus pentosaceus* CACC616 in Weaned Piglets

**DOI:** 10.3390/microorganisms11122890

**Published:** 2023-11-30

**Authors:** Soyeon Park, Jeongsup Song, Mi Ae Park, Hyun-Jun Jang, Seoyun Son, Dae-Hyuk Kim, Yangseon Kim

**Affiliations:** 1 Department of Research and Development, Center for Industrialization of Agricultural and Livestock Microorganisms, Jeongeup 56212, Republic of Korea; soyun8648@gmail.com (S.P.); jungsup717@cialm.or.kr (J.S.); mapark@cialm.or.kr (M.A.P.); 316wkd@cialm.or.kr (H.-J.J.); ssy9911@cialm.or.kr (S.S.); dhkim@cialm.or.kr (D.-H.K.); 2 Department of Molecular Biology, Institute for Molecular Biology and Genetics, Jeonbuk National University, Jeonju 54896, Republic of Korea; 3 Department of Bioactive Material Science, Jeonbuk National University, Jeonju 54896, Republic of Korea

**Keywords:** weaned piglets, calprotectin, gut microbiota, probiotic, *Pediococcus pentosaceus*

## Abstract

During weaning, piglets experience various stressor events that disrupt their gut microbiota and immune balance, decrease growth parameters, and increase mortality rates. In this study, we assessed the efficacy of *Pediococcus pentosaceus* CACC616 as a probiotic supplement. We characterized this strain and evaluated its effect on improving growth performance, modulating gut microbiota composition, and reducing noxious odor components in weaned piglets compared to a non-supplementary diet (control). During the 26-day period, 40 crossbred weaned piglets were randomly assigned to pens with 20 animals each in two groups: control and treatment groups with CACC616. On day 26, the treatment group exhibited a lower feed conversion ratio (FCR) and a significant alteration in gut microbial composition, correlating with improved growth parameters and gut health (*p* < 0.05). The treatment group also exhibited significantly reduced digestibility- and intestinal-environment-related noxious odor components (*p* < 0.05). The CACC616 strain effectively reduced pathogenic genera numbers, including *Campylobacter*, *Mogibacterium*, *Escherichia–Shigella,* and *Desulfovibrio* spp., with the treatment group exhibiting lower fecal calprotectin levels than the control group (*p* < 0.05). Overall, this study revealed that the functional probiotic CACC616 contributes to enhanced FCR and effectively modulates weaned piglets’ inflammation and intestinal microbiota.

## 1. Introduction

Weaning is a highly stressful event for piglets, leading to the main economic damage of pig farms [1,2]. During post-weaning, stress-induced gut microbiota dysbiosis in weaned piglets can result in growth depression, the long-term breakdown of the intestinal barrier functions, the induction of an inflammation state, and increased disease susceptibility. To prevent these problems, feed antibiotics have been used in post-weaning piglets to promote production, reduce odor gas, and prevent infections [2,3]. However, antibiotic misuse has generated and exponentially increased multidrug-resistant bacteria in humans and animals [4]. Probiotics, antimicrobial proteins, and bacteriophages are now the most promising and emerging antibiotic alternatives [5,6]. Utilizing dietary probiotic bacteria, including lactic acid bacteria (LAB), has proven effective in controlling the intestinal health of piglets [7,8].

LAB have been used as probiotics owing to their beneficial health and therapeutic effects. They can modulate immune function, synthesize and increase nutrient bioavailability, and defend against pathogenic microorganisms [9,10,11,12]. Among them, Lactobacilli have been mainly used as probiotics for increasing growth performance and health in livestock [12,13]. Furthermore, LAB applications contributed to stabilizing intestinal microbiota and reducing the production of odorous gas by regulating digestibility [14,15]. However, the studied probiotics for the feed supplements have been relatively limited to *Lactobacillus* and *Bifidobacterium* [16]. Therefore, there is a need to discover additional strains to broaden the application range of probiotics and enhance their applications for livestock. The genus *Pediococcus,* a member of the *Lactobacillaceae* family, is a Gram-positive, catalase-negative, facultatively anaerobic, rod-shaped LAB that has been studied as a probiotic supplement [16,17]. Dowarah et al. (2017) showed that supplementation with *P. acidilactici* FT28 promoted feed efficiency and increased the LAB population, resulting in intestinal health and relieving diarrheal symptoms in pigs [18]. However, limited data are available on the effects of diets with *P. pentosaceus* in weaned piglets, specifically regarding the correlation between intestinal microbial composition and beneficial effects such as enhanced growth performance and the reduced emission of fecal noxious gases. Therefore, we evaluated the effect of diets supplemented with *P. pentosaceus* CACC616 on the growth and blood parameters, intestinal microbiota composition, and noxious gas emissions in weaned piglets.

## 2. Materials and Methods

### 2.1. P. pentosaceus Isolation and Identification

*P. pentosaceus* CACC616 was isolated from fecal samples of pigs obtained from Republic of Korea. The samples were serially diluted 10-fold with sterile phosphate-buffered saline (PBS) and plated on de Man, Rogosa and Sharpe (MRS; Merck KGaA, Darmstadt, Germany) agar plates, followed by a 24 h incubation period at 35 °C. Through 16s rRNA sequencing (518F: 5’-CCAGCAGCCGCGGTAATAC-3’ and 805R: 5’-GACTACCAGGGTATCTAATC-3’), 462 isolates were isolated and identified. The acquired 16S rRNA sequences of the isolated strains were aligned with the NCBI GenBank database using BLAST (http://www.ncbi.nlm.nih.gov/BLAST/Blast.cgi, accessed on 16 August 2023) and compared to previously deposited 16S rRNA sequences.

### 2.2. Tolerance to Artificial Gastrointestinal Conditions

Probiotic candidates must be able to survive in the stomach and small intestine environments. Therefore, the tolerance evaluation of *P. pentosaceus* CACC616 was performed in a specific condition similar to the stomach (low pH) and the small intestine (bile salt) [19]. The *P. pentosaceus* CACC616 strain was cultured for 18 h in an MRS medium. *Lactobacillus rhamnosus* GG (ATCC 53103) was used as the positive control. The culture was transferred into sterile PBS with pH 2.5 and pH 7.2 (as a control) and incubated at 37 °C for 2 h. For the bile salt tolerance test, the culture was transferred into an MRS broth containing 0.3% oxgall and incubated at 35 °C for 8 h, followed by 10-fold serial dilutions for inoculation on the LAB Petrifilms and 24 h of cultivation at 35 °C. The results were expressed as bacterial strain colony-forming units (CFU), as enumerated using Petrifilm.

### 2.3. Adhesion Ability to Intestinal Epithelial Cells

The adhesion ability of the *P. pentosaceus* CACC616 strain to intestinal cells (HT-29 and IPEC-J2) was evaluated, as previously reported, with some modifications [19]. Cells were cultured in Dulbecco’s Modified Eagle’s Medium (DMEM; Gibco, Grand Island, NY, USA) supplemented with 10% heat-inactivated fetal bovine serum (FBS; HyClone, Logan, UT, USA) and 1% penicillin–streptomycin (Gibco) at 37 °C in a 5% CO_2_ atmosphere. The monolayer cells were prepared in a 24-well plate (SPL Life Science, Pocheon, Republic of Korea) at a density of 1 × 10^5^ cells/well of HT-29 and 1.5 × 10^4^ cells/well of IPEC-J2 cells. The pellet of *P. pentosaceus* CACC616 was harvested, suspended with DMEM, and adjusted to approximately 1.0 × 10^9^ CFU/mL (OD_600nm_ = 1.0). The prepared bacteria (approximately 1.0 × 10^8^ CFU/well) were inoculated on 24-well plates and incubated for 2 h at 37 °C under 5% CO_2_. Subsequently, all plates were washed three times with sterile PBS to remove nonattached bacterial cells, with the attached bacterial cells detached using 1% Triton X-100 (Sigma-Aldrich, St. Louis, MN, USA). The number of viable bacterial cells was enumerated on LAB Petrifilms (3M^TM^, St. Paul, MN, USA). *L. rhamnosus* GG was used as the control.

### 2.4. Hemolytic and Biogenic Amine (BA) Activities

To determine the hemolytic activity of the *P. pentosaceus* CACC616 strain, an overnight-grown culture was streaked on blood agar plates supplemented with 5% (*v/v*) sheep blood (MBcell, Seoul, Republic of Korea) and incubated at 37 °C for 24 h. After incubation, the hemolytic reaction was evaluated [20]. The BAs (putrescine, cadaverine, histamine, spermidine, and spermine) of the *P. pentosaceus* CACC616 strain were determined using a previously described procedure [20].

### 2.5. Antibiotic Susceptibility (Minimal Inhibitory Concentration, MIC)

The antibiotic susceptibility was tested using the commercial E-test^®^ strip (bioMerieux, Marcy-l’Etoile, France): ampicillin, vancomycin, gentamicin, kanamycin, streptomycin, erythromycin, clindamycin, tetracycline, and chloramphenicol. The fresh cultures were inoculated and spread on MRS agar plates, after which the antibiotic strips were deposited on the surface of the MRS plates and incubated at 37 °C for 24 h. The MIC cutoff values of the nine antibiotics were determined following the 2022 European Food Safety Authority (EFSA) guidelines [21].

### 2.6. Cell Culture and Treatment

RAW264.7 murine macrophage-like cells were cultured in DMEM medium supplemented with 10% FBS and 1% penicillin–streptomycin at 37 °C in a 5% CO_2_ atmosphere. The cells were subcultured and plated at 70–80% confluency, followed by transfer to six-well plates at 5 × 10^5^ cells/well and overnight culturing. *P. pentosaceus* CACC616 or *L. rhamnosus* GG (LGG) was grown overnight at 37 °C in MRS broth. Overnight-grown bacterial cultures were re-inoculated at 1/100 into fresh MRS and grown until the OD_600_ was 1.0 (>1 × 10^9^ CFU/mL). All bacteria were diluted to 1 × 10^7^ or 1 × 10^8^ CFU/mL in DMEM and inoculated into 6-well plates (Multiplicity of Infection, MOI 10 and 100). Six treatment groups were included: control group, nontreatment medium only; lipopolysaccharide (LPS) group, where cells were stimulated with 1 µg/mL of LPS (Enzo, Farmingdale, NY, USA); and probiotic groups, where cells were co-stimulated with LPS and *P. pentosaceus* CACC616 (Multiplicity of Infection, MOI 10 and 100) or the *L. rhamnosus* GG (probiotic control strain) for 4 h.

### 2.7. Quantitative Real-Time Reverse Transcription–Polymerase Chain Reaction (RT-PCR)

The total RNA was extracted from the LPS or LPS-stimulated RAW264.7 cells using a RNeasy Plus Mini Kit (Qiagen, Hilden, Germany) and then quantified using NanoDrop One (Thermo Scientific, Waltham, MA, USA). Reverse transcription was performed using AccuPower RT PreMix (Bioneer, Seoul, Republic of Korea). The resulting products were subjected to qRT-PCR performed using Bio-Rad CFX96 (BIO-RAD Laboratories, Hercules, CA, USA) with the following conditions: 95 °C denaturation for 5 min, followed by 40 cycles of 95 °C for 10 s, 60 °C annealing, and extension for 30 s, and melt curve to confirm the amplification specificity. Table S1 contains the target gene primer sequences. The relative amounts of these mRNAs were determined using the comparative (2^−ΔΔCT^) method, as previously described [22].

### 2.8. Experimental Design, Diet, and Feeding

All of the procedures used in this experiment were approved by the Institutional Animal Care and Use Committee of the Center for Industrialization of Agricultural and Livestock Microorganisms (approval #CIALM 2023-03). Forty weaned pigs (Duroc × Landrace × Yorkshire, 21 days of age at 7.8 ± 1.5 kg body weight (BW)) were used in a 26-day experiment. All pigs were allotted two dietary treatments based on a randomized complete block design, with gender and initial BW as blocks. *P. pentosaceus* CACC616 was grown in industrial medium (0.5% fructose, 2% yeast extract, 1% soy peptone, 0.5% Tween 80, 0.2% dipotassium phosphate, 0.5% sodium acetate, 0.2% ammonium citrate dibasic, 0.01% magnesium sulfate, 0.01% manganese sulfate, and pH 6.5) at 37 °C for 24 h. The culture was freeze-dried, and the feed additive was prepared to contain 4.8 × 10^11^ CFU/g. The dietary treatment was divided into three phases: Phase I (days 0–9, Purina neo smile 2), Phase II (days 10–20, Purina neo smile 3), and Phase III (days 21–26, Purina relay). Two groups were established: (1) basal diet as a negative (N) control (Purina neo smile 2–3-relay; (Cargill Agri Purina, Inc., Seongnam, Republic of Korea) and (2) CACC616, a basal diet supplemented with 0.5% of *P. pentosaceus* CACC616 (2.4 × 10^9^ CFU/g feed). During the 26-day trial period, all groups were provided ad libitum access to water and feed, and the room temperature was controlled at approximately 26 °C with 60 ± 5% humidity. Table S2 lists the total nutrient levels of the basal diets (Purina, Cargill).

BW was recorded individually on days 0 (D0), 13 (D13), and 26 (D26), and feed consumption was also documented daily by measuring the feed refused by each group. The average daily gain (ADG), average daily feed intake (ADFI), and feed conversion ratio (FCR = ADFI/FCR) were calculated for 0–9, 10–20, and 0–26 days of the trial. The number of weaning pigs with diarrhea per treatment was monitored and counted on days 0–13 and 14–26. Mortality was measured on day 26. Diarrhea and mortality rates were calculated using the following formula: (a) Diarrhea rate (%) = the total number of weaning pigs with diarrhea/the total number of weaning pigs × 100% and (b) Mortality rate (%) = the total number of dead weaning pigs/the total number of weaning pigs × 100%.

### 2.9. Chemical Analysis

On day 26 (D26), blood samples were collected from all groups. The hematological and biochemical parameters measured in plasma samples were white blood cells (WBCs), red blood cells (RBCs), hemoglobin (Hb), hematocrit (Hct), mean corpuscular volume (MCV), mean corpuscular hemoglobin (MCH), mean corpuscular hemoglobin concentration (MCHC), and platelets. In contrast, those in serum samples were total protein, blood urea nitrogen (BUN), creatinine, cortisol, glucose, aspartate aminotransferase (AST), alanine aminotransferase (ALT), and cholesterol.

For chemical analysis, the diet and fecal samples were dried at 300 °C for 1.5 h. All diet and fecal samples were analyzed for crude protein, fiber, fat, ash, and moisture following the Association of Official Analytical Chemists methods [23].

### 2.10. Fecal Odorous Gas Analysis

Odorous compounds such as volatile fatty acids (VFAs) and volatile organic compounds (VOCs) were measured using gas chromatography [24]. Here, 30 g of the fecal sample was subjected to thermal desorption (330 °C for 4 h) under a 100 mL min^−d^ flow of N_2_. Preconditioning at 230 °C for 30 min for subsequent use was tested as sufficient and applied for all tubes. The odor-casing materials (VFA and VOC) were qualitatively analyzed using a gas chromatography–pulsed flame photometric detector (Varian 450-GC, Bruker, Billerica, MA, USA).

### 2.11. Enzyme-Linked Immunosorbent Assay (ELISA)

Fecal calprotectin is a noninvasive biomarker of intestinal inflammation in humans and animals [25]. Representative fecal samples were obtained from each group on D26. The samples were extracted, diluted, and determined using a commercial Porcine Calprotectin ELISA kit (MyBioSource, San Diego, CA, USA) following the manufacturer’s procedures.

Serum IgG and IgM antibody responses were measured using a double-antibody sandwich ELISA, as previously described, with some modifications [26,27]. Checkerboard titration was employed to determine the optimal capture, serum antibody, and enzyme conjugate concentrations [28,29]. The optimal concentrations of capture, serum, and enzyme-conjugated antibodies were selected to measure anti-pig IgG and anti-pig IgM antibody responses. Briefly, 96-well microplates (Nunc immune MaxiSorp, ThermoFisher Inc., Waltham, MA, USA) were coated at 4 °C overnight with 100 μL/well of sheep anti-pig IgG (1.0 μg/mL; Bethyl Laboratories, Montgomery, TX, USA) or goat anti-pig IgM (2.5 μg/mL; Novus Biologicals, LLC., Littleton, CO, USA) in carbonate buffer (0.05 M, pH 9.6). The wells were washed three times with PBS containing 0.05% Tween 20 (PBS-T) and blocked with 2% FBS powder in PBS-T for 2 h at 37 °C. The serum samples of each group were diluted to 1:200 and added to the blocked wells. After 1 h of incubation, the wells were washed with PBS-T and added with HRP-conjugated goat anti-pig IgG (1:40,000 dilution; Bethyl Laboratories) or goat anti-pig IgM (1:60,000 dilution; Bethyl Laboratories) secondary antibodies. After incubation, the wells were washed three times to remove unbound secondary antibodies. The enzyme reaction was then inhibited by adding 100 μL/well HRP-substrate 3,3’,5,5’-tetramethylbenzidine (TMB; Surmodics Inc., Eden Prairie, MN, USA) to each well and incubating for 3 min. The enzyme reaction was terminated by adding 100 μL/well of 0.5 M H_2_SO_4_. The OD was measured at 450 nm using a spectrophotometer (TECAN, Männedorf, Switzerland).

### 2.12. mRNA Sequencing, Data Processing, and Metagenome Analysis

Eighty rectal swabs (BD BBL^TM^ CultureSwab^TM^, Sparks, MD, USA) were collected from 40 piglets repeatedly at D0 and D26. All sequencing, data processing, and analyses were conducted by LAS, Inc. (Gimpo, Republic of Korea). Microbial gDNA was extracted from rectal swab samples following previously reported methods [30]. The extracted gDNA was quantified using a DropSense96 spectrophotometer (Trinean, Gentbrugge, Belgium). The metagenomic library was prepared using an MGIEasy universal library prep kit (MGI, Shenzen, China) and sequenced through the MGISEQ-2000 system with 300-bp paired-end reads. An analysis of the full data was performed using the Quantitative Insights Into Microbial Ecology 2 (QIIME2) program using the SILVA database v.132 (13 December 2017). Then, the sequences were processed with a Divisive Amplicon Denoising Algorithm 2 to remove noise such as paired-end sequences, dereplicate them, merge read pairs, and filter PCR chimeras. Furthermore, the taxonomic classification process was performed using QIIME2 software v. 2022.2.0.

The relative abundance at the phylum, family, and genus levels was compared between the CACC616 and control groups. Furthermore, eight genera were analyzed and visualized in R software using a violin plot. The principal coordinate analysis (PCoA) was performed and visualized using R (v. 4.2.3) via R studio interface (v. 2023.06.2-561) to evaluate differences in the fecal microbiota between the two groups. Statistical analyses of bacterial taxonomic composition between groups were performed using PERMANOVA. Alpha diversity indexes (Chao1 and Shannon) were calculated and compared between the CACC616 and control groups.

### 2.13. Statistical Analyses

All results were analyzed using one-way analysis of variance (ANOVA) using the post hoc Tukey test in GraphPad version 5.0 software (GraphPad Software, Inc., San Diego, CA, USA). For all experiments, *p* < 0.05 was considered statistically significant. Data were expressed as the mean ± standard error of the mean (SEM). Relative abundance, alpha, and beta diversity were analyzed using the R program (v.4.2.3) (http://www.R-project.org, accessed on 13 December 2017) and plyr (v.1.8.8), reshape2 (v.1.4.4) and phyloseq package (v.1.42.0). Alpha diversity and relative abundance were analyzed using the Kruskal–Wallis test. Post normalization, PCoA was performed using the Bray–Curtis dissimilarity index via vegan (v.2.6-4). OUT/genus was used to present the violin plot (v.0.4.0) and was examined using one-way ANOVA, followed by the Tukey–Kramer test. All data of significance were set at *p* < 0.05.

## 3. Results

### 3.1. Potential for Survival in the Gastrointestinal Environment of P. pentosaceus CACC616

**Figure 1:**
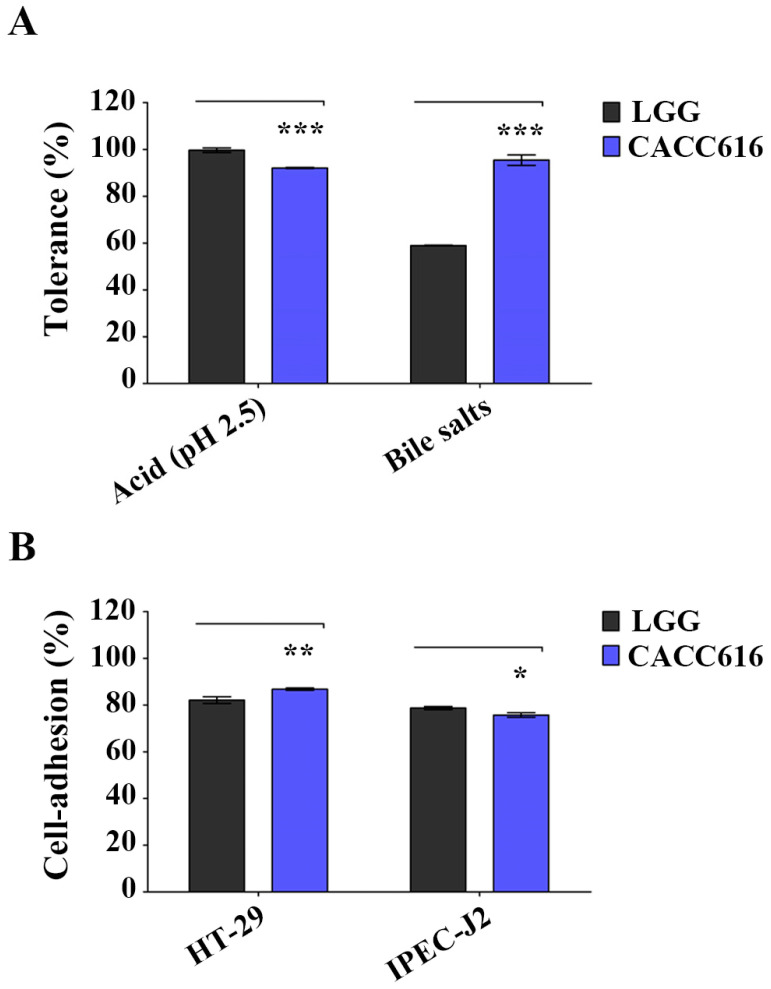
Potential probiotic properties of *Pediococcus pentosaceus* CACC616. (**A**) Tolerance to acid and bile salts of the CACC616 strain and (**B**) bacterial adhesion ability to human colorectal adenocarcinoma cell line (HT-29) and intestinal porcine enterocyte cell line (IPEC-J2) cells. Data are presented as means ± SD. * *p* < 0.05, ** *p* < 0.01, *** *p* < 0.001.

*P. pentosaceus* CACC616 was tested for its tolerance to acid and bile salt conditions (low pH) and its adhesion ability to intestinal cells (HT-29 and IPEC-J2). The strain showed tolerance at pH 2.5 for 2 h (88.4%) and 0.3% oxgall for 8 h (95.7%) (Figure 1A). The ability of bacteria to adhere to intestinal cell surfaces is related to colonization and persistence in the gastrointestinal tract [31]. The *P. pentosaceus* CACC616 strain exhibited an efficient adhesion ability to HT-29 and IPEC-J2 cell surfaces (86.6% and 75.1%, respectively; Figure 1B).

### 3.2. Safety Assessment of P. pentosaceus CACC616

The safety assessment of *P. pentosaceus* CACC616 was conducted by testing for bacterial antibiotic susceptibility, BA production ability, and hemolytic activity. Table 1 contains the results of the MIC values for antibiotic susceptibility of the CACC616 strain against nine assayed antibiotics. Five MIC values against ampicillin, gentamicin, erythromycin, clindamycin, and chloramphenicol were lower than the cutoff values stipulated in the EFSA’s technical guidelines [21]. *P. pentosaceus* CACC616 was sensitive to ampicillin and clindamycin and intermediate to erythromycin (Table 1).

The total major BAs of *P. pentosaceus* CACC616 were rarely produced: putrescine (5.19 ppm), cadaverine (2.59 ppm), spermidine (11.5 ppm), and spermine (5.45 ppm) (Table 2). According to the EFSA’s guidelines, histamine is the most toxic. In our results, histamine was not produced by *P. pentosaceus* CACC616. The CACC616 strain did not show hemolytic activity after inoculating it on an agar plate containing 5% sheep blood (Figure S1). Collectively, *P. pentosaceus* CACC616 was confirmed safe for use in probiotic applications based on the testing of BA production and hemolytic activity.

### 3.3. Immune Modulation of P. pentosaceus CACC616 on LPS-Stimulated Inflammatory Cytokine Expression

**Figure 2:**
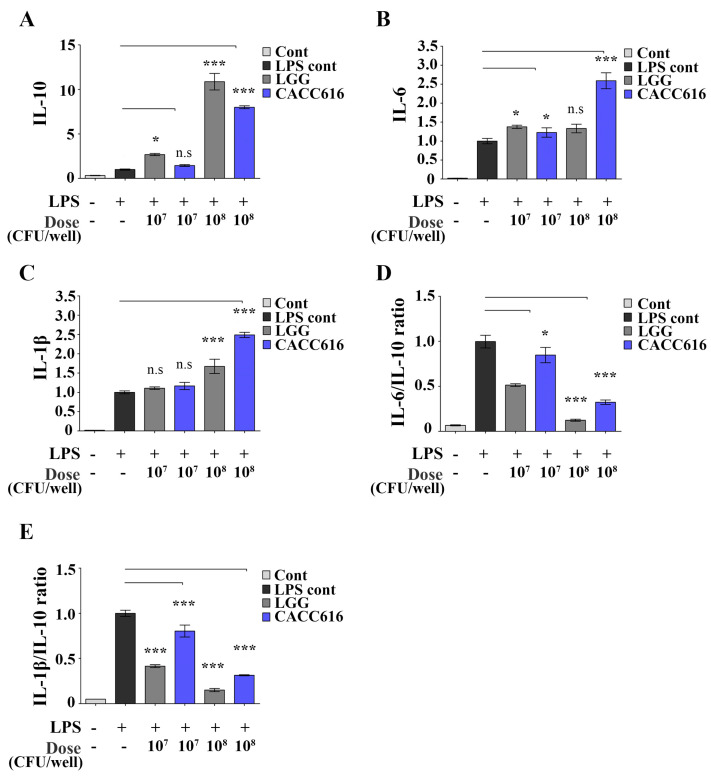
Effect of *Pediococcus pentosaceus* CACC616 on cytokine IL−1β, IL−6, and IL−10 expression and ratios in LPS-stimulated RAW264.7 cells. The effect on anti- and pro-inflammatory indicators such as cytokine (**A**) IL−10, (**B**) IL−6, and (**C**) IL−1β expressions and (**D**) IL−6/IL−10 and (**E**) IL−1β/IL−10 ratios in RAW264.7 cells. Data are presented as the means for the three independent replicates (mean ± SD). * *p* < 0.05, *** *p* < 0.001.

The pro- and anti-inflammatory cytokines IL−1β, IL−6, and IL−10 play essential roles in immune regulation due to monocyte maturation [32]. RAW264.7 cells were treated with different doses of *P. pentosaceus* CACC616 (1 × 10^7^ or 1 × 10^8^ CFU/dose) to evaluate their immune-modulating ability (Figure 2). The strain of CACC616-treated RAW264.7 cells significantly increased LPS-induced IL−6 and IL−1β expression (1.2- to 2.6-fold and 1.2- to 2.5-fold increase, respectively; *p* < 0.05) compared to the LPS-treated control (Figure 2B,C). Notably, anti-inflammatory cytokines, such as IL−10, were also dramatically increased in the CACC616-treated RAW264.7 cells compared to the LPS-treated control (1.4- to 8.0-fold increase, *p* < 0.05) (Figure 2A). The IL−6/IL−10 and IL−1β/IL−10 ratios were significantly lower in the CACC616- or LGG-treated RAW264.7 cells compared to the LPS-treated control group (Figure 2D,E). Therefore, *P. pentosaceus* CACC616 played a bifunctional role in regulating the expression of pro- and anti-inflammatory cytokines in LPS-stimulated RAW264.7 cells.

### 3.4. Effect of Potential Probiotic Supplementation on Weaned Piglets

To evaluate the effect of potential probiotic supplementation on the growth of weaned pigs in terms of BW, the average daily weight gain, ADFI, and FCR were measured. The initial BWs of the CACC616 and control groups were 7.7 ± 0.3 kg and 7.3 ± 0.3 kg, and the final BWs were 16.9 ± 0.6 kg and 16.5 ± 0.6 kg, respectively (Table 3). There was no significant difference in the initial and final BWs between the CACC616 and control diet groups. However, none of the groups showed clinical symptoms, including diarrhea and mortality. The CACC616 group displayed lower ADFI and FCR than the control group (*p* > 0.05). The CACC616-supplemented diet had greater crude protein and fiber digestibility values than the control diet (Table S3), although the difference was insignificant.

### 3.5. Effect of Potential Probiotic Supplementation on Fecal Noxious Gas Emissions

Table 4 contains the results of the effect of the CACC616-supplemented diet on fecal noxious gas emissions in weaned pigs. The CACC616 diet group exhibited significantly lower butyric and valeric acid concentrations than the control group. Significant differences were not observed in the concentrations of acetic, propionic, isobutyric, or isovaleric acids. Major odorous gases such as phenol, p-cresol, hydrogen sulfide, and methyl mercaptan generated in pig farms pollute the environment. The CACC616 diet group showed lower fecal phenol, p-cresol, skatol, and methyl mercaptan emissions than the control group (*p* < 0.05). Additionally, the fecal indol of the CACC616 diet group was substantially reduced.

### 3.6. Effect of Potential Probiotic Supplementation on Hematological Parameters and Immune Modulation

**Figure 3:**
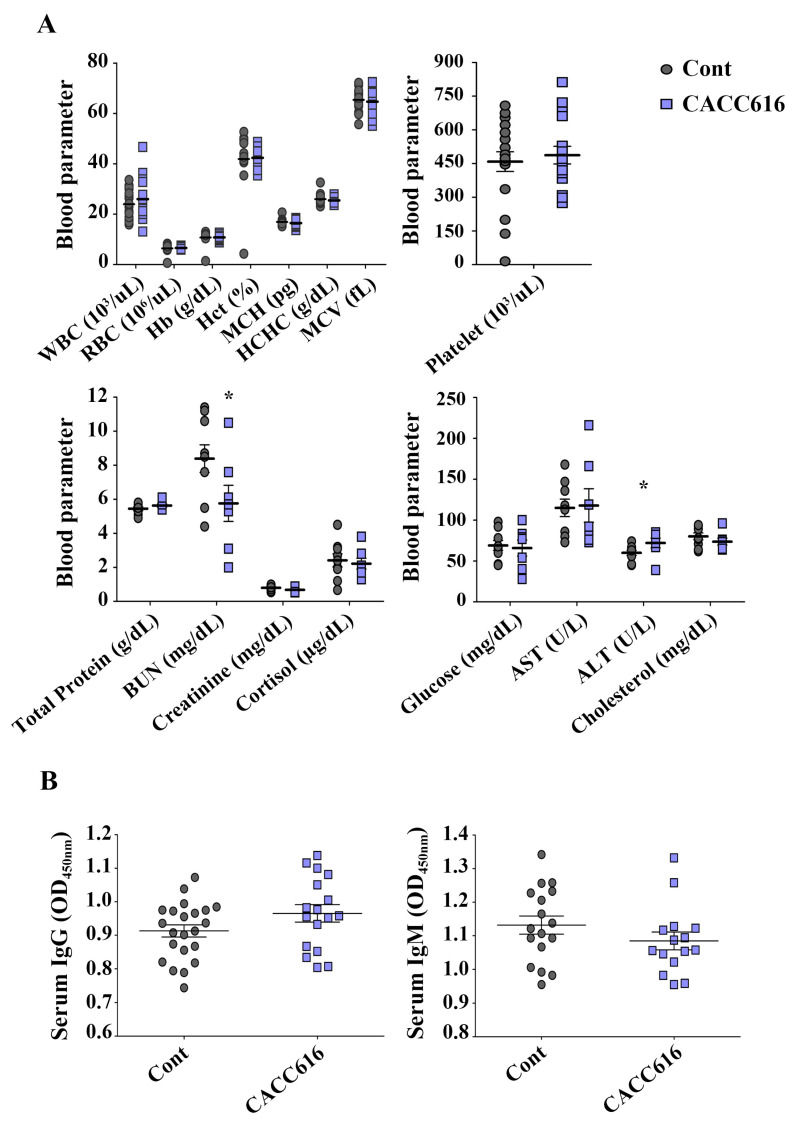
Effect of dietary *P. pentosaceus* CACC616 supplementation on blood parameters and serum antibodies. (**A**) Variations in blood parameters (*n* = 8) and (**B**) serum anti-pig IgG and IgM levels (*n* = 20) according to diet with CACC616. All values are expressed as the mean ± SEM. *p*-values were determined using the Student’s paired *t*-test, where * *p* < 0.05. Control: non-supplementary diet; probiotic composition: normal diet supplemented with *P. pentosaceus* CACC616. WBC: white blood cell; RBC: red blood cell; Hb: hemoglobin; Hct: hematocrit; MCH: mean corpuscular hemoglobin; MCHC: mean corpuscular hemoglobin concentration; MCV: mean corpuscular volume; BUN: blood urea nitrogen; ALT: alanine aminotransferase; AST: aspartate transaminase.

Figure 3 shows the effect of *P. pentosaceus* CACC616 on the hematological parameters of the weaned pigs. The results of the hematological parameters revealed no statistically significant differences during the experiment for all groups except for the parameters BUN and ALT. The CACC616 group had higher WBCs and cortisol values than the control group (*p* > 0.05). Moreover, digestibility-related parameters such as BUN and cholesterol tended to be lower for the CACC616 group than for the control group (Figure 3A). Notably, the BUN value of the CACC616 group showed a 1.5-fold reduction compared to the control group (8.4 ± 0.3; *p* < 0.05).

**Figure 4:**
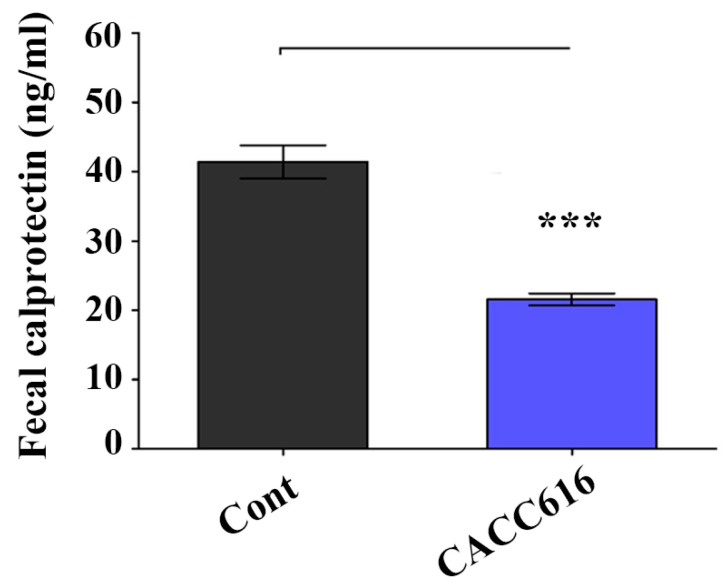
Comparison of fecal calprotectin among weaned piglets in the *P. pentosaceus* CACC616 and control groups. The concentration of fecal calprotectin between the two groups. All values are expressed as the mean ± SEM (*n* = 18). *p*-values were determined using the Student’s paired *t*-test where *** *p*  < 0.05. Control: non-supplementary diet; probiotic composition: normal diet supplemented with *P. pentosaceus* CACC616.

To analyze the effects of the probiotic *P. pentosaceus* CACC616 on humoral immunity levels, the concentrations of intestinal inflammation indicators were evaluated by detecting serum immunoglobulins (IgG and IgM) and fecal calprotectin in pigs (Figure 3B). While the serum total IgG levels tended to increase in the CACC616-supplemented group compared with the control group (*p* > 0.05), the serum IgM levels tended to decrease (*p* > 0.05). Furthermore, CACC616 treatment resulted in a 50% reduction in the calprotectin concentration (19.5 ± 2.1 mg/mL) compared to the nontreated control groups (39.4 ± 6.3 mg/mL; *p* < 0.001) (Figure 4).

### 3.7. Effect of Dietary CACC616 on the Fecal Microbiota Composition of Weaned Piglets

The effect of *Pediococcus*-based dietary supplementation on the microbiota composition in weaned piglets was investigated. Taxonomic analysis revealed that 160 fecal samples yielded a total of 25,667,568 reads. The reading sequence number ranged from 40,582 to 544,679 (mean: 320,845; median: 333,555). The sequences were processed and clustered into 959 operational taxonomic units (OTUs). Table 5 contains the relative abundance (%) of the fecal microbiota of the CACC616 and control groups at the genus levels. Both groups’ core taxa at the genus level were observed in the top 19 genera with the highest relative abundance (the abundance cutoff was set at 0.1%). On day 0, *Psychrobacter* (44.2% and 39.1%), *unknown_Lachnospiraceae* (9.7% and 13.4%), and *Ruminococcaceae UCG* (14.1% and 12.3%) were the most prevalent groups in the CACC616 and control groups, respectively. After 26 days (D26), these groups underwent some changes: *Prevotella* (16.6%), *Ruminococcaceae UCG* (14.1%), *Escherichia–Shigella* (10.5%), and *Lactobacillus* (9.1%) were mainly found in the control group, whereas *Prevotella* (15.0%), *Ruminococcaceae UCG* (14.0%), *Lactobacillus* (9.4%), and *Escherichia–Shigella* (7.9%) were mainly found in the CACC616 group.

**Figure 5:**
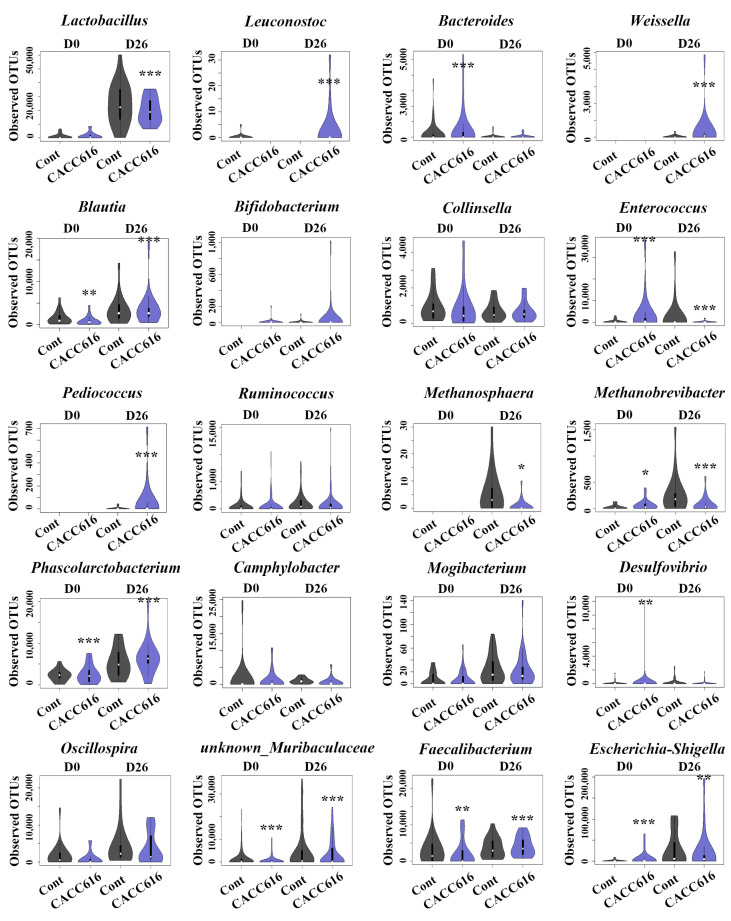
Changes in the fecal microbiota associated with diet supplementation with *P. pentosaceus* CACC616. The violin plot represents the bacteria’s relative abundance at the genus level. In this plot, the white dot indicates the median value of observed OTUs, the black bar is the interquartile range, and the horizontal width of the violin plot indicates the density of the data along the Y axis. All values are expressed as the mean ± SEM (*n* = 20). * *p* < 0.05, ** *p* < 0.01, *** *p* < 0.001. Control: non-supplementary diet; probiotic composition: normal diet supplemented with *P. pentosaceus* CACC616.

Figure 5 shows the abundance of specific bacterial genera. Twenty OTUs/genera were compared, revealing that *Leuconostoc*, *Weissella, Blautia, Pediococcus, Phascolarctobacterium*, *Fusobacterium*, and *unknown_Muribaculaceae* had higher abundance in the CACC616 group than in the control group (*p* < 0.05), but *Lactobacillus*, *Enterococcus*, *Methanosphaera*, *Methanobrevibacter*, *Faecalibacterium*, and *Escherichia–Shigella* showed lower abundance (*p* < 0.05). The *Campylobacter*, *Mogibacterium*, and *Desulfovibrio* abundances were lower in the CACC616 group than in the control group (*p* > 0.05). Additionally, we analyzed the relative abundance of the intestinal microbiota, which exceeded more than 0.1% of the microbiota at the phylum and family levels (Figure S2). The intestinal microbiota was dominated by the phyla Firmicutes and Bacteroidetes irrespective of a diet with CACC616, which contained 55.5–55.6% and 32.4–33.0% of the abundance, respectively (Figure S2), at D26. The major families of the CACC616 group were *Lactobacillaceae* (6.3%), *Enterobacteriaceae* (5.3%), and *Rikenellaceae* (4.8%) at D26. However, the dominant families of the control group were *Enterobacteriaceae* (6.8%), *Lactobacillaceae* (6.0%), and *Rikenellaceae* (5.4%). CACC616 treatment tended to reduce the families of *Enterobacteriaceae* and *Rikenellaceae* compared to their relative abundance in the control group (*p* > 0.05).

The microbial diversity of the fecal samples was compared between the CACC616 and control groups (Figure S3). Beta diversity was performed using the Bray–Curtis dissimilarity method, and the distance between the two groups was displayed using PCoA. A distinct separation was not observed between the microbiota composition of the CACC616 and control groups (Figure S3A). Furthermore, the alpha diversity did not differ in either group (Chao1, *p* = 0.388; Shannon, *p* = 0.660) (Figure S3B).

## 4. Discussion

Weaning stress in piglets can alter the intestinal microbiome environment, resulting in a low digestion rate, the high occurrence of diarrhea, an imbalanced intestinal microbiota, and an unmodulated immune system [33,34]. In this study, the *P. pentosaceus* CACC616 strain was isolated and characterized, after which its potential probiotic was evaluated. Its ability to improve growth performance, intestinal composition, and immune modulation in weaned piglets when incorporated as dietary supplements was also assessed. *P. pentosaceus* CACC616 showed excellent probiotic properties compared to the LGG control, as well as bacterial safety for using the probiotics such as the producing ability of biogenic amines, antibiotic resistance, and hemolytic ability. Lee et al. (2023) observed the immune modulation effects of *P. pentosaceus* as immunostimulants, elevating the mRNA levels of cytokines IL-10, IL-6, and IL-1β [35,36]. These studies corroborate our findings; therefore, *P. pentosaceus* CACC616 can be applied as a potential probiotic.

Although our study found that *P. pentosaceus* CACC616 treatment resulted in increased ADFI and FCR in weaned piglets after 26 days, how supplementation with CACC616-based functional probiotics improved weaned piglet growth remains unclear. In this study, several reasons can be proposed: the improved digestibility, such as decreasing the BUN and cholesterol in the serum level; the reduction in digestibility-associated odor gas, such as p-cresol, indole, and skatole; and the elevating abundance of beneficial gut microorganisms, such as *Leuconostoc*, *Blautia*, *Bifidobacterium*, *Ruminococcus*, and *Weissella* spp.

*P. pentosaceus*-supplemented diets significantly improved the growth performance of pigs and reduced fecal noxious gas and diarrhea incidence [14,17]. *P. pentosaceus* can modulate immunity and ameliorate gut inflammation by maintaining gut epithelial integrity [36,37]. Our results corroborated these findings, as fecal calprotectin, an indicator of intestinal inflammation, was significantly reduced in the CACC616 group.

The variation in blood parameters can indicate digestibility-related metabolism and health [38]. The total protein and BUN levels are strongly related to the digestion and absorption of protein, particularly the value of BUN, a major end product of protein metabolism in pigs [38,39]. As digestibility increases, fewer substrates are available for intestinal microbial fermentation, hence reducing fecal noxious gas emissions in the environment [40]. This prior research corroborates our finding of reduced BUN and noxious gas levels in weaned piglets.

Para (p)-cresol, a uremic toxin, negatively affects pigs’ growth performance [41]. In our study, the CACC616 group exhibited a markedly reduced fecal p-cresol level. While the only notable difference in the CACC616 group was observed in FCR, a significant increase in *Blautia* abundance was also observed (Table 1 and Figure 5). This result aligns with previous findings indicating that *Blautia*, a core genus in the pig gut microbiota, is positively correlated with daily weight gain and feed efficiency [42,43].

Gut microbiota-associated odor compounds (p-cresol, indole, and skatole) were reduced in the CACC616 group (Table 4). Skatole is a malodorous compound generated through the deamination and decarboxylation of tryptophan by *Bacteroidetes* [44]. We observed that the *Bacteriode* abundance was reduced in both the CACC616 and control groups at D26 (D0 vs. D26, *p* < 0.05). *Oscillibacter* abundance correlates negatively with indole levels and positively with skatole levels [45]. In the present study, the CACC616 treatment resulted in reduced *Oscillibacter* abundance and fecal compound levels (indole and skatole) compared to the control (Figure 5). *Mogibacterium*, *Desulfovibrio*, *Ruminococcus*, *Clostridium sensu stricto*, and *Campylobacter* exhibited marked correlations with hydrogen sulfide [46,47]. Decreased *Mogibacterium*, *Desulfovibrio*, *Ruminococcus,* and *Campylobacter* populations were observed in the CACC616 group compared to the control group. *Methanobrevibacter* spp. and *Methanosphaera* spp. were found to be abundant in the small and large intestines, colon, and feces of pigs [43,47], and they positively correlated with fiber digestibility [44]. Although feed supplemented with *P. pentosaceus* CACC616 did not affect nutrient digestibility in pigs, alterations in gut microbiome composition were observed (Figure 5). Additionally, dysentery in pigs was correlated with pathogenic bacteria such as *Campylobacter*, *Mogibacterium*, *Escherichia–Shigella,* and *Desulfovibrio* spp., with reduced abundance levels observed in the treatment group. The protective effect of *P. pentosaceus* against pathogenic bacteria has been reported [17,47]. These studies suggest that CACC616 may have contributed to the improved health conditions of piglets’ gut microbiota.

## 5. Conclusions

In this study, an increase in the abundance of *P. pentosaceus* was confirmed in the gut microbiota (Figure 5). Several external and internal factors influence this colonization in pigs. Both our study and previous findings support the beneficial effects of feed additives, demonstrating their impact on gut inflammation and the reduction of noxious gas emissions due to changes in the intestinal environment resulting from *P. pentosaceus* CACC616 administration. However, there are few studies on the beneficial effects of *P. pentosaceus* on microbiome-based growth performance and intestinal health in pigs. Overall, our findings expand existing knowledge and uncover potentially beneficial applications of probiotic supplements that will benefit pig farming.

## Figures and Tables

**Table 1:** Antibiotic susceptibility profiles of the *Pediococcus pentosaceus* CACC616 strain.

Antibiotic	MIC (µg/mL) ^1^
Ampicillin	**≥3.0**
Vancomycin	0.0
Gentamicin	≥32.0
Kanamycin	0.0
Streptomycin	≥384.0
Erythromycin	1.0
Clindamycin	**≥0.094**
Tetracycline	≥24.0
Chloramphenicol	≥6.0

^1^ MICs lower than EFSA cutoff values (µg/mL) in bold; MIC: minimum inhibitory concentrations. Control: non-supplementary diet. Probiotic composition: normal diet supplemented with *P. pentosaceus* CACC616.

**Table 2:** Biogenic amine-producing abilities of the *Pediococcus pentosaceus* CACC616 strain.

BA *-Producing Ability (ppm)					Total Concentration (ppm)
Putrescine	Cadaverine	Histamine	Spermidine	Spermine	
5.19	2.59	ND	11.5	5.45	24.73

* ND: not detected. Control: non-supplementary diet. Probiotic composition: normal diet supplemented with *P. pentosaceus* CACC616.

**Table 3:** Effect of dietary *Pediococcus pentosaceus* CACC616 supplementation on growth performance in weaned piglets.

Items (0–26 d)	Control	*Pediococcus pentosaceus* CACC616
Initial BW (kg)	7.7 ± 0.3	7.3 ± 0.3
Final BW (kg)	16.9 ± 0.6	16.5 ± 0.6
ADG (g)	354 ± 13	354 ± 12
ADFI (g)	587 ± 26	558 ±24
FCR	1.70 ± 0.07	1.63 ± 0.06

All values are expressed as mean ± SEM (*n* = 20). Control: non-supplementary diet; probiotic composition: normal diet supplemented with *P. pentosaceus* CACC616. ADG: average daily gain; ADFI: average daily feed intake; FCR: feed conversion ratio.

**Table 4:** Effect of dietary *Pediococcus pentosaceus* CACC616 supplementation on fecal noxious gases.

Items	Control	*Pediococcus pentosaceus* CACC616
VFAs		
Acetic acid	3727.5 ± 689.0 ^a^	3190.0 ± 754.0 ^a^
Propionic acid	14,978.6 ± 668.6 ^a^	15,119.5 ± 2205.5 ^a^
Isobutyric acid	2212.2 ± 96.9 ^a^	1994.0 ± 679.0 ^a^
Butyric acid	8759.0 ± 255.2 ^a^	4102.5 ± 748.5 ^b^*
Isovaleric acid	2391.6 ± 83.8 ^a^	1429.7 ± 259.4 ^a^
Valeric acid	5096.0 ± 80.8 ^a^	2641.7 ± 567.9 ^b^**
VOCs		
Phenol	3.9 ± 0.1 ^a^	10.0 ± 0.6 ^b^**
p-cresol	526.7 ± 21.8 ^a^	281.0 ± 59.0 ^b^*
Indole	1.4 ± 1.4 ^a^	0.3 ± 0.0 ^a^
Skatole	52.0 ± 6.0 ^a^	21.9 ± 5.3 ^b^*
Hydrogen sulfide	1109 ± 594.2 ^a^	192.7 ± 24.3 ^a^
Methyl mercaptan	171.7 ± 12.4 ^a^	59.7 ± 15.4 ^b^***

All values are expressed as mean ± SEM (*n* = 3). Control: non-supplementary diet; probiotic composition: normal diet supplemented with *P. pentosaceus* CACC616; VFAs: volatile fatty acids; VOCs: volatile organic compounds. ^a,b^ Means were used to indicate a statistically significant difference (* *p* < 0.05, ** *p* < 0.01, *** *p* < 0.001).

**Table 5:** Intestinal microbial composition of the CACC616 and control groups at the genus level.

Genus	D0		D26	
	Control	CACC616	Control	CACC616
*Psychrobacter*	39.10	44.15	0.33	0.01
*Unknown_Lachnospiraceae*	13.39	9.74	5.45	5.71
*Ruminococcaceae UCG*	12.28	14.13	14.11	14.01
*Prevotella*	6.25	2.95	16.58	14.98
*[Eubacterium] coprostanoligenes group*	4.34	3.45	2.68	1.74
*Uncultured_bacterium_Porphyromonadaceae*	4.06	3.82	6.82	5.71
*Unknown_Ruminococcaceae*	3.12	2.35	1.29	1.15
*Rikenellaceae RC9 gut group*	2.49	3.94	6.48	5.64
*Prevotellaceae NK3B31 group*	2.43	0.80	5.31	7.09
*Unknown_Prevotellaceae*	2.30	0.84	2.13	2.80
*Uncultured_bacterium_Muribaculaceae*	2.06	1.14	1.81	3.54
*Ruminococcaceae NK4A214 group*	1.81	2.06	3.30	3.24
*Phascolarctobacterium*	1.66	1.93	2.07	2.79
*Alloprevotella*	1.66	0.89	3.36	4.54
*Clostridium sensu stricto 1*	1.47	1.08	3.27	4.36
*Lactobacillus*	0.72	1.17	9.14	9.43
*Escherichia–Shigella*	0.69	5.34	10.45	7.94
*Unknown_Veillonellaceae*	0.01	0.01	3.09	3.27
*Agathobacter*	0.00	0.00	2.18	1.91
<Others (0.1%)	0.16	0.20	0.15	0.14

All values are expressed as mean ± SEM (*n* = 20). Control: non-supplementary diet; probiotic composition: normal diet supplemented with *P. pentosaceus* CACC616.

## Data Availability

The data presented in this study are available in the presented article or Supplementary Materials.

## Supplementary Materials

The following supporting information can be downloaded at: https://www.mdpi.com/article/10.3390/microorganisms11122890/s1. Figure S1: Hemolytic activity. Figure S2: Effect of dietary *P. pentosaceus* CACC616 supplementation on intestinal microbiota composition in weaned piglets. Figure S3: Intestinal microbiota richness and difference in microbiome structure on alpha and beta-diversity at genus level. Table S1: Primer sequences used in this study. Table S2: Total nutrient levels of the basal diets for pigs (air-dry basis; %). Table S3: Effect of dietary *P. pentosaceus* CACC616 supplementation on nutrient digestibility.
