# Supplementary material for: Assessing the Probiotic Effects of Pediococcus pentosaceus CACC616 in Weaned Piglets
Source: Microorganisms. 2023 Nov 30;11(12):2890. doi: 10.3390/microorganisms11122890 (PMC10746064; doi:10.3390/microorganisms11122890)
Supplement: Supplementary file 1 [file microorganisms-11-02890-s001.zip › microorganisms-2724230-supplementary.pdf]

Supplementary Material
Assessing the probiotic effects of *Pediococcus pentosaceus* CACC616 in weaned piglets

Park et al.

Table S1. Primer sequences used in this study.

Target gene	Forward primer	Reverse primer
IL-1β	5'-CCTGGGCTGTCCTGATGAGAG-3'	5'-CGGGAAAGACACAGGTA-3'
IL-6	5'-AGTCCGGAGAGGAGACTTCA-3'	5'-ATTTCCACGATTTCCCAGAG-3'
IL-10	5'-TGGGTTGCCAAGCCTTATCG-3'	5'-TTCAGCTTCTCACCCAGGGA-3'
GAPDH	5'-GGCCTTCCGTGTTCCTAC-3'	5'-TGCCTGCTTCACCACCTTC-3'

Control: non-supplement diet. Probiotic composition: normal diet supplemented with *P. pentosaceus* CACC616.

Table S2. Total nutrient levels of the basal diets for pigs (air-dry basis; %)

Items	Phase I (D0–9)	Phase II (D10–20)	Phase III (D21–26)
Digestible energy, Mcal/kg	3.40	3.40	3.60
Crude protein, %	20.0 ± 0.2	19.7 ± 0.2	17.9 ± 0.0
Crude fat, %	7.05 ± 0.2	5.36 ± 0.1	6.3 ± 0.3
Crude fiber, %	1.8 ± 0.1	2.5 ± 0.1	2.4 ± 0.2
Crude ash, %	5.8 ± 0.1	5.3 ±0.1	5.3 ± 0.1
Lysine, %	1.35	1.20	1.10
Calcium, %	0.50	0.50	0.50
Phosphorus, %	0.80	0.70	0.70
Alanine, %	0.95	0.91	0.85
Arginine, %	1.16	1.16	1.08
Cystine, %	0.54	0.43	0.41
Glutamic acid, %	3.33	3.42	3.15
Glycine, %	0.81	0.84	0.68
Histidine, %	0.57	0.49	0.46
Isoleucine, %	0.79	0.76	0.68
Leucine, %	1.72	1.55	1.53
Lysine, %	1.73	1.40	1.37
Methionine, %	0.42	0.35	0.30
Phenylalanine, %	0.93	0.90	0.84
Proline, %	1.19	1.19	1.12
Valine, %	0.99	0.92	0.79
Tyrosine, %	0.00	0.56	0.55
Tryptophan, %	0.26	0.20	0.19

Table S3. Effect of dietary *P. pentosaceus* CACC616 supplementation on nutrient digestibility

**Groups**	**Control**	***P. pentosaceus* CACC616**
Moisture	72.8 ± 2.0	76.7 ± 0.5
Dry matter	27.2 ± 2.0	23.3 ± 0.5
Crude protein	26.8 ± 1.3	29.8 ± 0.8
Crude fiber	7.3 ± 0.3	8.3 ± 0.5
Crude fat	10.7 ± 1.5	10.3 ± 1.6
Crude ash	11.9 ± 0.3	12.0 ± 0.4

All values are expressed as mean ± SEM (n = 5). Control: non-supplement diet. Probiotic composition: normal diet supplemented with *P. pentosaceus* CACC616.

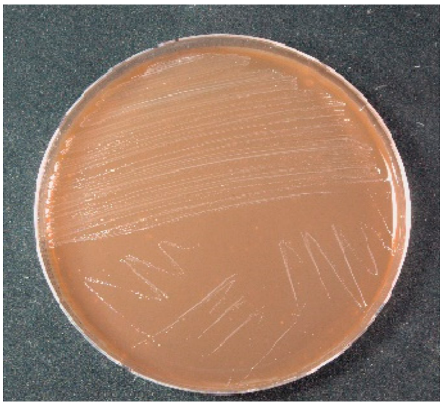

Figure S1. Hemolytic activity.

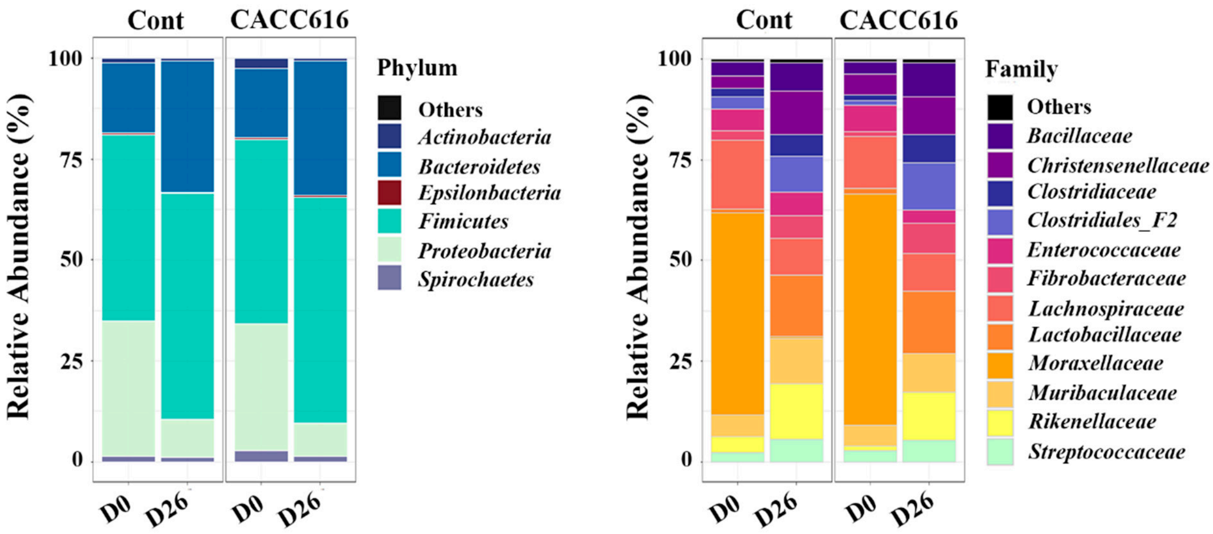

Figure S2. Effect of dietary *P. pentosaceus* CACC616 supplementation on intestinal microbiota composition in weaned piglets. Relative abundance bar plot at the phylum and family level. All values are expressed as mean ± SEM (n = 20). Control: non-supplement diet. Probiotic composition: normal diet supplemented with *P. pentosaceus* CACC616.

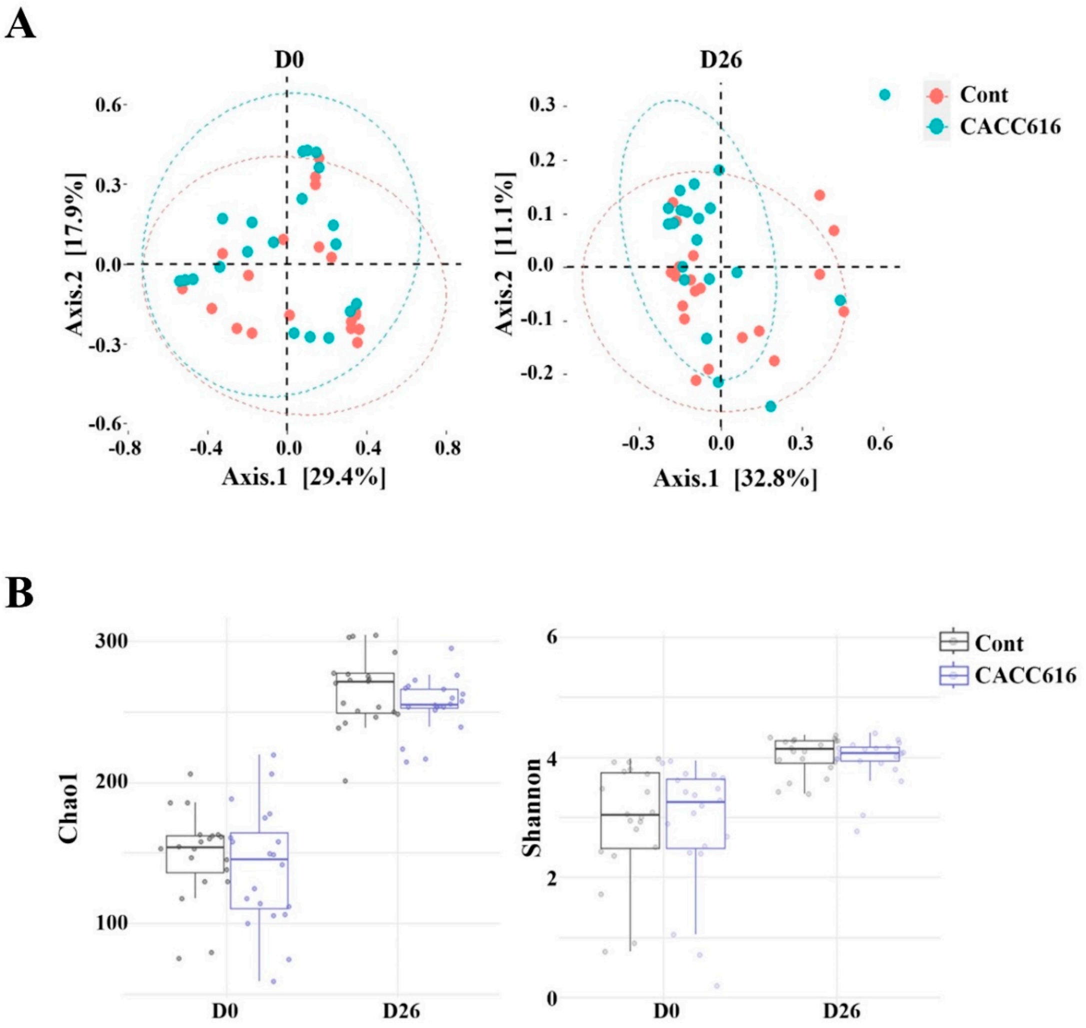

Figure S3. Intestinal microbiota richness, difference of microbiome structure on alpha and beta-diversity at genus level. (A) comparison of beta-diversity based on Principal coordinate analysis (PCoA), (B) alpha-diversity index (Chao1 and Shannon) between CACC616 and control groups. All values are expressed as mean ± SEM (n = 20). Control: non-supplement diet. Probiotic composition: normal diet supplemented with *P. pentosaceus* CACC616.
